# Increasing frequency and spatial extent of concurrent meteorological droughts and heatwaves in India

**DOI:** 10.1038/s41598-017-15896-3

**Published:** 2017-11-14

**Authors:** Shailza Sharma, Pradeep Mujumdar

**Affiliations:** 1 Department of Civil Engineering, Indian Institute of Science, Bangalore, 560012 India; 2 Divecha Center for Climate Change, Indian Institute of Science, Bangalore, 560012 India

## Abstract

The impacts of concurrent droughts and heatwaves could be more serious compared to their individual occurrence. Meteorological drought condition is generally characterized by low rainfall, but impact of such an event is amplified with simultaneous occurrence of heatwaves. Positive feedback between these two extremes can worsen the rainfall deficit situation to serious soil moisture depletion due to enhanced evapotranspiration. In this study, the concurrence of meteorological droughts and heatwaves is investigated in India using Indian Meteorological Department (IMD) high resolution gridded data over a period of 60 years. Significant changes are observed in concurrent meteorological droughts and heatwaves defined at different percentile based thresholds and durations during the period 1981–2010 relative to the base period 1951–1980. There is substantial increase in the frequency of concurrent meteorological droughts and heatwaves across whole India. Statistically significant trends in the spatial extent of droughts are observed in Central Northeast India and West Central India; however, the spatial extent affected by concurrent droughts and heatwaves is increasing across whole India. Significant shifts are identified in the distribution of spatial extent of concurrent drought and heatwaves in India compared to the base period.

## Introduction

India has witnessed severe droughts in the recent past and heatwaves have also been occurring frequently across many parts of the country. Indian monsoon droughts of 1982, 1987, 2002 and 2009 had an adverse impact on agricultural production causing huge economic losses and extrapolations of epochal nature of ocean-atmosphere system suggest frequent monsoon droughts during 2020–2049^1^. Significant long term increasing trends are observed in the frequency of heatwaves over India during recent 50 years (1961–2010), with 2001–2010 being the warmest decade for the country^2^. Increase in the frequency, total duration and maximum duration of heatwaves is observed over central and north-western parts of the country^3^. Intensification of longer duration and highly frequent heatwaves is projected in India, which can increase the heat stress and mortality rate^4^. Attempts have also been made to understand the principle mechanisms of droughts and heatwaves. Severe droughts in India have always been accompanied by El-Niño events, yet all the El-Niño events have not resulted in severe droughts^5^. The years with maximum number of heatwave days are found to be preceded by warm ENSO years^6^. In a recent study, two types of heatwaves identified based on observed patterns and statistical analysis of maximum temperature variability in north-central India and coastal eastern India, are found to be associated with anomalous blocking over North Atlantic Ocean and anomalous baroclinic Matsuno-Gill response to the anomalous cooling in Pacific respectively^7^.

Droughts^8^ and extreme summer heatwaves^9–11^ have increased over many regions across the globe. Positive feedback between droughts and heatwaves has been considered as a relevant factor for past heatwaves and temperature extremes^12–14^. A combination of low rainfall (meteorological drought) and abnormally high temperatures (heatwaves) can have more negative consequences compared to their individual occurrence. Concurrent droughts and heatwaves have received a great attention in recent studies^15–18^. Individual occurrence of droughts^19–21^ and heatwaves^3,7^ in India is extensively studied, but their joint occurrence is not yet explored.

In the present study, concurrence of meteorological droughts and heatwaves in India is investigated. A heatwave is defined as the period of consecutive days with conditions hotter than normal^22^. Number of consecutive days with maximum temperature above 90^th^ percentile is generally taken as three or five^23^ for defining a heatwave. Heatwaves of ≥8 days are observed in north, northwest, central and northeast Peninsula on an average per season^2^. Therefore, 3-day, 5-day and 10-day heatwave events defined above 85^th^, 90^th^ and 95^th^ percentiles of maximum daily temperature are considered in this study. Heatwave Magnitude Index daily (HWMId)^24^, which combines the duration and magnitude of heatwaves is used to examine the strength and spatial extent of historical heatwave events in India. Since the primary interest of this study is in accumulated rainfall deficits to address meteorological drought conditions, the Standardized Precipitation Index^25^ (SPI) based definition of meteorological drought with an averaging period of 1 month is used. Droughts are classified as moderate (SPI < −1.3), severe (SPI < −1.6) and extreme (SPI < −2) following the World Meteorological Organisation guidelines for using SPI^26^.

## Results and Discussion

### Heatwaves

**Figure 1:**
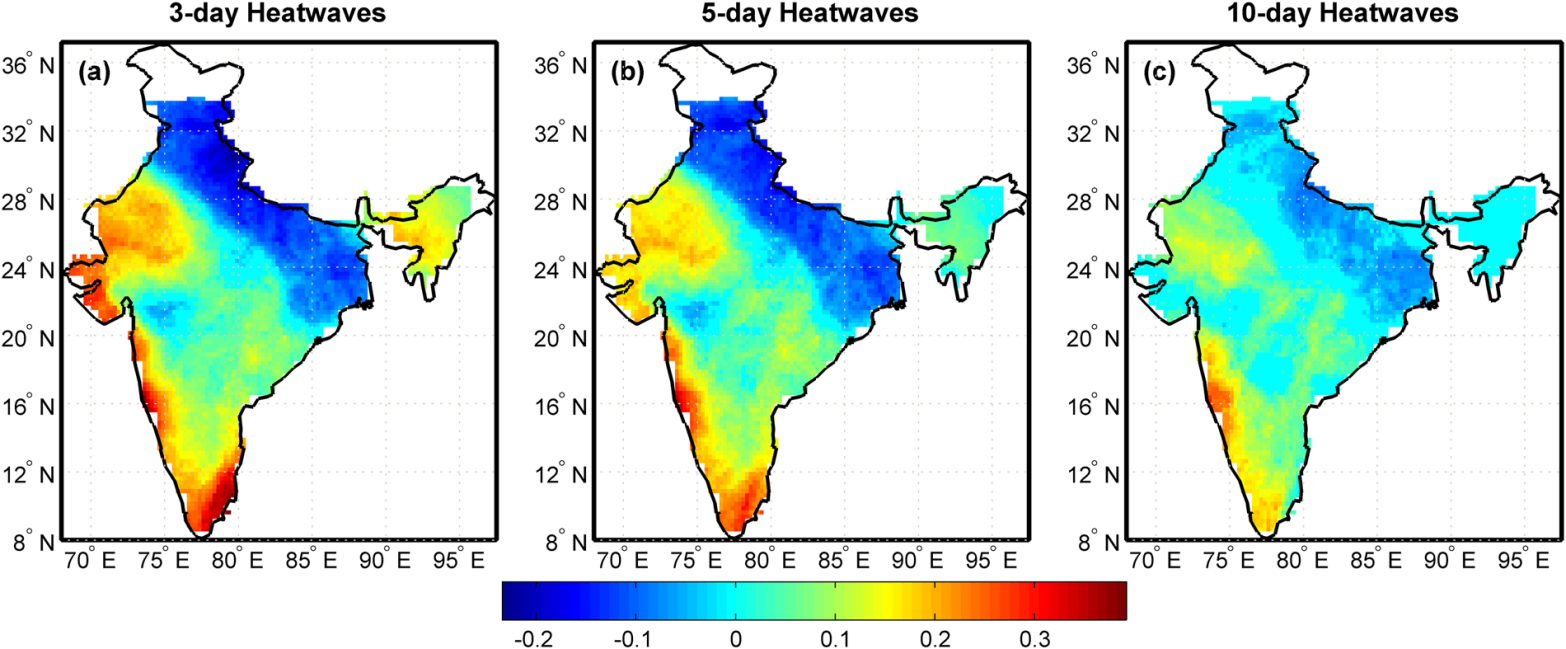
Trends in frequency of 85^th^ percentile heatwaves over India during the period 1951–2010 for different durations. (**a**) 3-day heatwaves (**b**) 5-day heatwaves and (**c**) 10-day heatwaves. Trends are detected using Mann-Kendall test and the magnitude of trend is quantified using Sen’s slope method at 95% significance level. This figure is plotted in Matlab R2014a (Version 8.3.0.532, URL: https://in.mathworks.com).

Analysing historical extremes is important to plan and prepare for mitigating associated impacts. Statistically significant trends in the frequency of 85^th^ percentile heatwaves of different durations (3-day, 5-day and 10-day) at 5% significance level, over a period of 60 years (1951–2010) are presented in Fig. 1. Non-parametric Mann-Kendall trend test^27^ is applied to detect trends in heatwave events and grid-wise slope of linear trends is computed using Sen’s slope method^28^. Increasing trends in heatwaves at 85^th^ percentiles are more pronounced in northwest India, in some parts of southwest India (Western Ghats) and southeast India (Eastern Ghats) for all the considered durations. Trends in 85^th^ percentile 3-day heatwaves are more pronounced in Northwest India (Rajasthan and Gujarat), Western Ghats, Tamil Nadu, and North-eastern States. Slight increase in the frequency of 3-day heatwaves is also observed along Eastern Ghats and in some regions of West Central India (Telangana, Chhattisgarh, Maharashtra and Madhya Pradesh) (Fig. 1a). Spatial area affected by 3-day and 5-day 85^th^ percentile heatwaves is almost same with slight difference in the magnitude of trends, particularly in north-eastern states (Fig. 1a,b). 10-day 85^th^ percentile heatwave events are prominent in Rajasthan, Western Ghats, Eastern Ghats and in some regions of Telangana, Chhattisgarh, Maharashtra and Madhya Pradesh (Fig. 1c). Results for heatwaves defined above 90^th^ and 95^th^ percentiles are presented in supplementary information (Figures S1 and S2).

**Figure 2:**
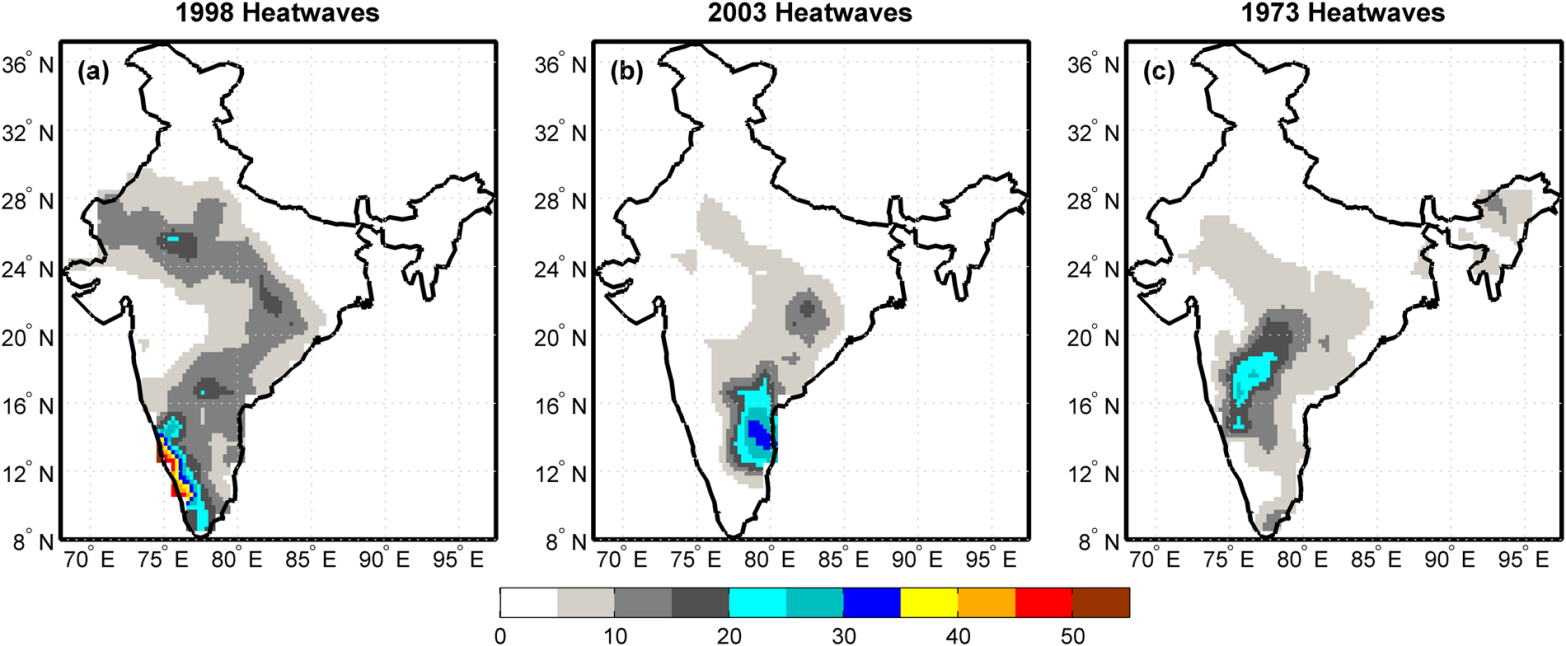
Major heatwaves in India during 1951–2010. The maps show the spatial distribution of HWMId for (**a**) 1998 heatwaves with maximum HWMId 55.8 at the grid 12.5 N, 76.5E (**b**) 2003 heatwaves with maximum HWMId 33.5 at the grid 14.5 N, 78.5E and (**c**) 1973 heatwaves with maximum HWMId 28.8 at the grid 16.5 N, 75.5E. This figure is created in Matlab R2014a (Version 8.3.0.532, URL: https://in.mathworks.com).

Historical heatwave events of India during the period 1951–2010 are ranked based on the peak of HWMId and the spatial extent (percentage of India exceeding a fixed HWMId level) in a particular year. Top five heatwave events are listed in Table S1 and Table S2 based on peak and spatial extent respectively. The year 1983 is ranked 1 based on the highest index value of 75.1, but the spatial extent affected by 1983 heatwaves was small compared to other heatwave events. The heatwaves of 1998 had the largest spatial extent among all heatwave events during 1950–2010, and thus ranked 1 based on the spatial extent. The spatial extent of 1998 heatwaves estimated with HWMId matches reasonably well with the observations^6^. The spatial distribution of HWMId for three years of major heatwaves is shown in Fig. 2. The time series and duration of these heatwaves are shown in Figure S3, for locations with highest HWMId.

### Meteorological Droughts

**Figure 3:**
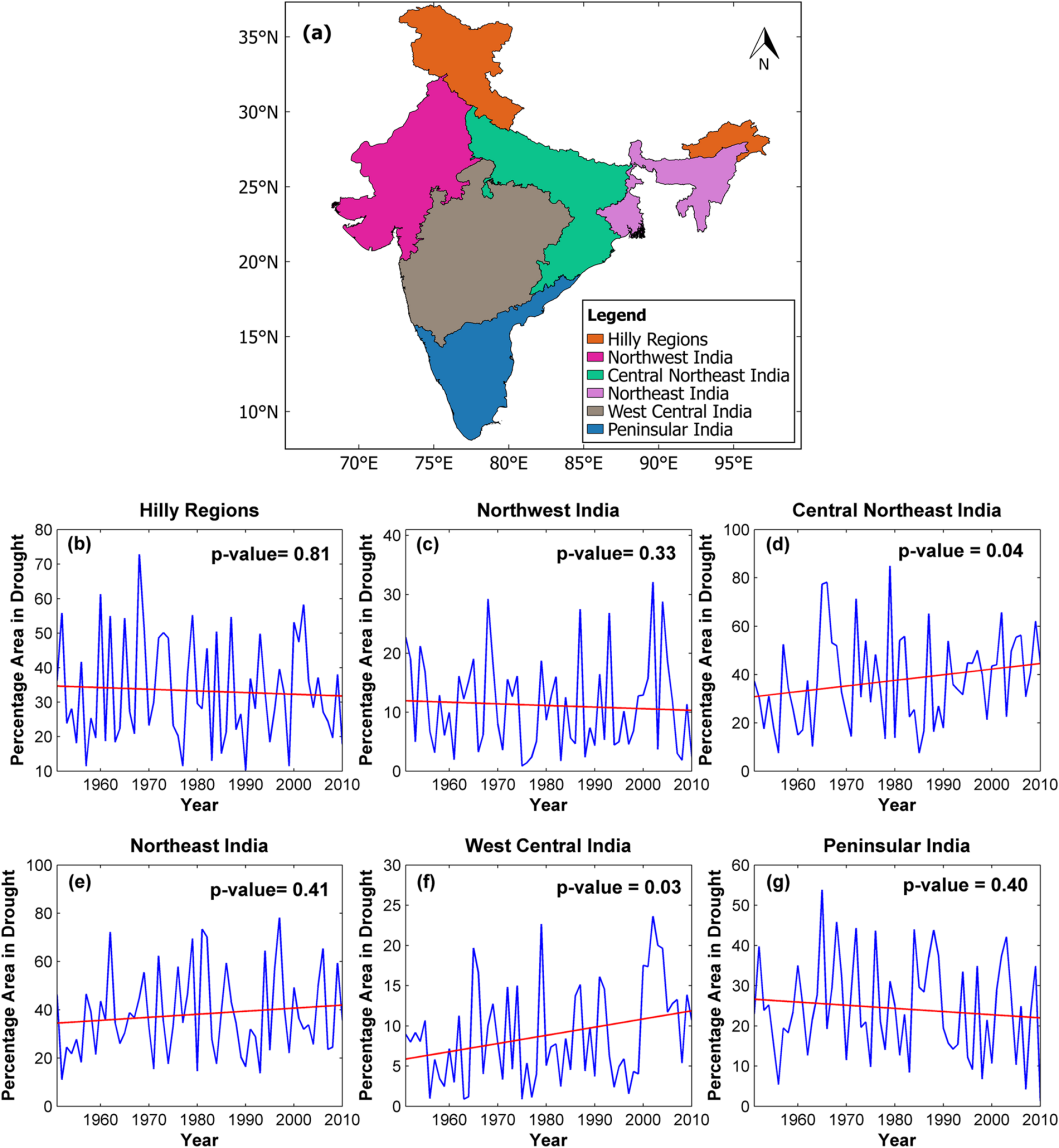
Percentage area in meteorological droughts (**a**) Meteorologically homogeneous regions of India and (**b**–**g**). Percentage area of different homogeneous regions in moderate droughts defined below SPI < −1.3. Mann-Kendall trend test results in terms of p-values are also shown here. Statistically significant trends are observed in the spatial extent of droughts from 1951 to 2010 for West Central India and Central Northeast India. The map of IMD homogeneous regions in this figure is prepared in QGIS (Version 2.14.0 ‘Essen’, free and open source geographic information system, QGIS Development Team (2016), URL: http://changelog.qgis.org/en/qgis/version/2.14.0/) and all other plots are generated using Matlab R2014a (Version 8.3.0.532, URL: https://in.mathworks.com).

Yearly spatial extent affected by metrological droughts is extracted for whole India (Figure S4a–c), but no trends are identified with Mann-Kendall trend test (Table S3). India is divided into five homogeneous regions based on rainfall characteristics and association of Indian summer monsoon rainfall with regional and global circulation^29^. Northeast hills, Jammu & Kashmir mountain regions were not considered in this study. The five IMD homogeneous regions and hilly regions (Northeast hills, Jammu & Kashmir mountain regions) are presented in Fig. 3a and yearly spatial area affected by moderate droughts defined below the threshold of SPI < −1.3 for these regions is presented in Fig. 3b–f. Monotonically increasing trends in the spatial extent of droughts are observed in Central Northeast India and West Central India. Significant decline in the monsoon rainfall of Indo-Gangetic plains^30^, and an increase in the percentage area of plains of India affected by moderate droughts^31^ were observed in earlier studies. Our results on individual occurrence of moderate droughts are consistent with these findings.

### Frequency of Concurrent Droughts and Heatwaves

**Figure 4:**
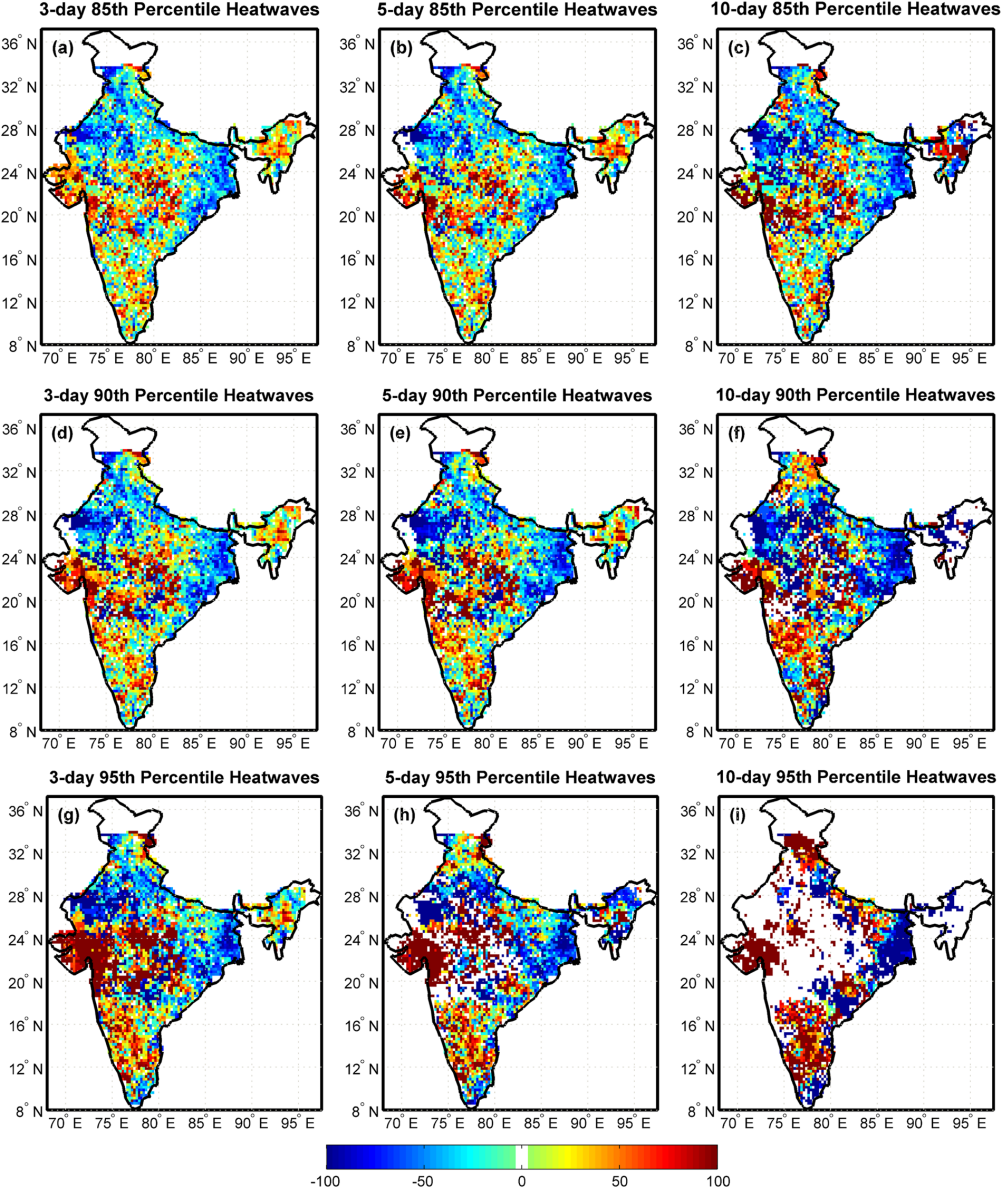
Percentage changes in concurrent moderate droughts defined below SPI < −1.3 and heatwaves during 1981–2010 with respect to the base period 1951–1980. Droughts with longer and severe heatwaves have increased much more than droughts with shorter duration and less severe heatwaves. Droughts and heatwaves are increasing across whole India and notably decreasing in Rajasthan and West Bengal. The maps are prepared in Matlab R2014a (Version 8.3.0.532, URL: https://in.mathworks.com).

Changes in the frequency of concurrent droughts and heatwaves are evaluated in this study. Available data is divided into two equal halves, each covering a period of 30 years. Percentage changes in concurrent extremes are based on the difference in number of events in 1981–2010 relative to the base period 1951–1980 divided by the total number of events. Percentage changes in concurrence of moderate droughts defined below SPI < −1.3 and heatwaves defined above different thresholds (85^th^, 90^th^ and 95^th^ percentiles) for durations (3-day, 7-day and 10-day) are presented in Fig. 4. Increase in concurrent moderate droughts and heatwaves is visible in patches across whole India; however, Central India, North-eastern States, South India and Gujarat are affected the most. Concurrent moderate droughts and 10-day 90^th^ percentile heatwaves have increased in Himachal Pradesh, Punjab, Gujarat, Central India and Peninsular India (Fig. 4f). Considerable decrease in concurrent extremes is observed in Rajasthan and West Bengal. Rajasthan, Madhya Pradesh, Maharashtra and some north eastern states show no changes in the concurrence of moderate droughts and longer and severe 10-day 95^th^ percentile heatwaves (Fig. 4i). Results of changes in the concurrence of severe droughts (SPI < −1.6) and extreme droughts (SPI < −2) with heatwaves are shown in Figures S5 and S6 respectively. An increase is observed in the concurrence of droughts and heatwaves across whole India compared to the base period 1951–1980 for all the combinations of droughts and heatwaves. Concurrence of droughts with longer and severe heatwaves is increasing in Gujarat, Central India and Peninsular India compared to short duration and less severe heatwaves.

### Spatial Extent of Concurrent Droughts and Heatwaves

**Figure 5:**
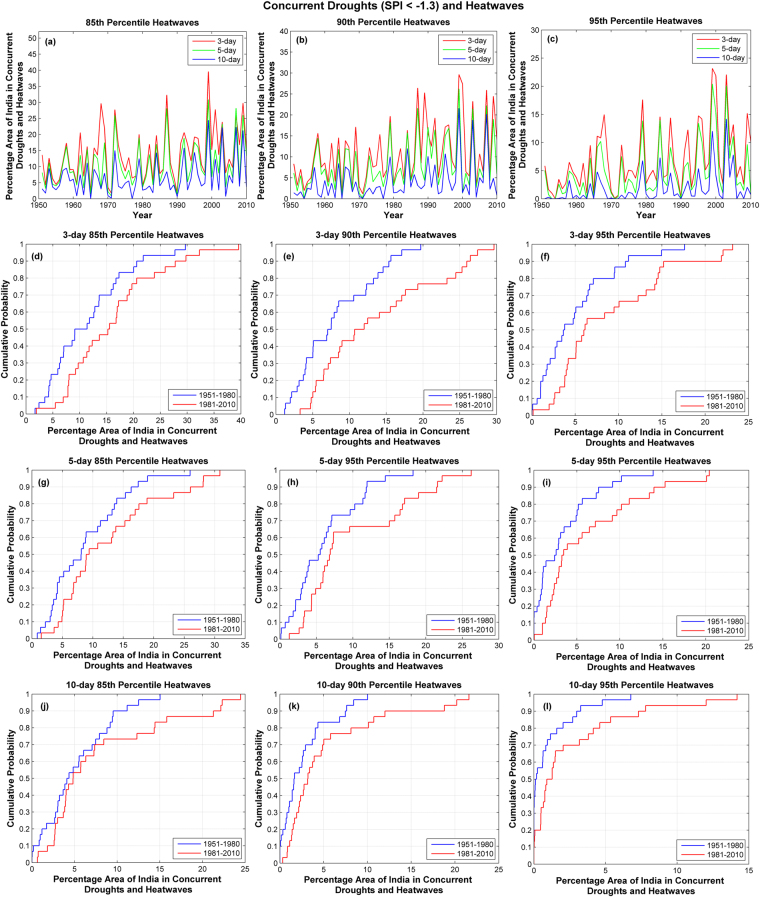
Spatial area of India in concurrent moderate droughts defined below SPI < −1.3 and heatwaves. Percentage area of India in concurrent moderate droughts (SPI < −1.3) and (**a**) 85^th^ percentile heatwaves (**b**) 90^th^ percentile heatwaves and (**c**) 95^th^ percentile heatwaves. Empirical CDFs of area affected by concurrent droughts (SPI < −1.3) and heatwaves for the periods 1951 to 1980 in blue and 1981 to 2010 in red (**d**–**i**). Significant departure is observed in CDFs during the period 1951 to 1980 compared to the base period for all combinations and divergence is highest in upper tail of 10-day heatwaves. These figures are generated using Matlab R2014a (Version 8.3.0.532, URL: https://in.mathworks.com).

Area affected by different combinations of concurrent droughts and heatwaves is extracted for every year to characterize the impact of such extremes. Spatial extent extracted for different combinations of moderate droughts (SPI < −1.3) and heatwaves is displayed in Fig. 5a–c. Increasing trends are observed in spatial extent of these extremes (Figure S7). Trend test results are presented in Table S4. Significant departure is observed in empirical CDFs of concurrent moderate droughts and heatwaves (Fig. 5d–l) between 1951–1980 and 1981–2010. Shifts in the upper tail of CDFs are more pronounced in the concurrence of droughts with 10-day heatwaves. Comparison of the empirical CDFs suggests that the spatial extent during the period 1981–2010 has longer right tail compared to that during the base period 1951–1980. This indicates that events affecting large spatial area are more in number in recent years compared to the base period. Spatial extent and empirical CDFs of concurrence of severe and exterme droughts with heatwaves are presented in Figures S8 and S9.

The two sample Kolmogorov-Smirnov (KS) test is commonly used for evaluating differences in the distributions. Cramér-von Mises test^32–34^ is known to be powerful for the case of different shapes of the distribution^35^, while Anderson-Darling test^36^ gives more weightage to the tails compared to the KS test. Statistical tests are sensitive to outliers; therefore box plots are used to detect outliers in yearly spatial extent of concurrent droughts and heatwaves (Figure S10). Most of the data is free of outliers, except for the 10-day heatwaves defined at 90^th^ and 95^th^ percentile. These outliers are retained in the data as they have strong influence in sociological terms. They are especially important in this study to enquire about the nature of recent extremes with respect to those during the base period. More information on accommodation of outliers in relation to probability models can be found in Barnett and Lewis^37^. The spatial extent of concurrent extremes from one year to the next can be assumed to be independent as the sample autocorrelations are found to be insignificant (Figure S11). Therefore two sample statistical tests can be applied to detect the shifts in the distribution.

The two sample Kolmogorov-Smirnov test failed to detect changes in the distribution of spatial extent of concurrent droughts and heatwaves. Both the Cramér-von Mises test and the Anderson-Darling test confirm that the distribution of spatial extent of India in concurrent droughts and heatwaves is different during 1981–2010 compared to the base period 1951–1980 at 5% significance level. Results of both these tests are presented in Table 1 to corroborate the conclusions.

**Table 1:** Two sample test results for the distributions of yearly spatial extent of India in concurrent moderate droughts (SPI < −1.3) and heatwaves.

Heatwaves	Cramér-von Mises		Anderson-Darling	
	p-value	Conclusion about CDFs	p-value	Conclusion about CDFs
3-day 85^th^ Percentile	0.0469	Different	0.0401	Different
3-day 90^th^ Percentile	0.1049	Identical	0.0040	Different
3-day 95^th^ Percentile	0.0494	Different	0.0123	Different
5-day 85^th^ Percentile	0.0100	Different	0.0157	Different
5-day 90^th^ Percentile	0.0348	Different	0.0178	Different
5-day 95^th^ Percentile	0.0397	Different	0.0121	Different
10-day 85^th^ Percentile	0.1084	Identical	0.3506	Identical
10-day 90^th^ Percentile	0.0287	Different	0.0197	Different
10-day 95^th^ Percentile	0.0221	Different	0.0080	Different

## Conclusions

Analysis of concurrent droughts and heatwaves over India during 1951–2010 is presented in this study, with a view to quantify the changes in their frequency and spatial extent. Establishing cause and effect relationship between the two extremes is beyond the scope of present work. Our results of individual analysis of extremes show that the heatwaves are increasing in Northwest India, Peninsular India, Northeast India, in some parts of West Central India and decreasing in Hilly regions and Central Northeast India. We further observe that 1983 heatwaves were strongest in terms of magnitude, while 1998 heatwaves had the largest spatial extent among all the heatwaves during the period 1950–2010. Meteorological droughts are seen to be increasing in Central Northeast India and West Central India. Concurrence of droughts and heatwaves has substantially increased during 1981–2010 relative to the base period 1951–1980. Increasing trends are observed in the spatial area affected by concurrent droughts and heatwaves. Significant changes in the distributions of spatial extent during the two periods are found using statistical tests.

One potential limitation of our approach is the restrictive definition of heatwaves based only on duration for examining changes in concurrent droughts and heatwaves. Considering other characteristics such as intensity and magnitude can improve the understanding of impacts of heatwaves. This study can be further extended by using model simulated or satellite driven soil moisture datasets to make the analysis more robust. Inclusion of soil moisture in the analysis will help in providing an understanding of the feedbacks between droughts and heatwaves.

## Methods

### Data

Indian Meteorological Department (IMD) high resolution gridded (0.25° latitude × 0.25° longitude) daily rainfall^38^ and high resolution gridded (1^0^ latitude × 1^0^ longitude) daily temperature^39^ datasets have been used in this study. Resolution of variables is different, so temperature data is re-gridded to the scale of rainfall using bilinear interpolation. More information on the adequacy of bilinear interpolation for smoothly varying variables by National Centre for Atmospheric Research (NCAR) can be found at https://climatedataguide.ucar.edu/climate-data-tools-and-analysis/regridding-overview. Based on data availability at all the grids, a common period of 60 years (1951–2010) is chosen. Six months (May to October) are considered in this study to cover most of the high temperature and low rainfall extreme events in summer and monsoon seasons. Total grids used in this study are 4316, which spread across whole India except the topmost part (Jammu & Kashmir) of India.

### Extraction of heatwaves and drought

Extremes of both the variables are identified based on peaks over threshold method. Heatwaves are defined as consecutive extreme hot days (3, 5 and 10 days) above different thresholds (85^th^, 90^th^ and 95^th^ percentile) of daily maximum temperature. To identify the strength and spatial extent of major heatwave events, we estimate the Heatwave Magnitude Index daily (HWMId) which combines the magnitude and duration of heatwaves. The computation of this index is done in open source statistical programming language ‘R’ using HWMId function of ExtRemes package^40^. HWMId is defined as the maximum magnitude of heatwaves in a year, where heatwave is a period of 3 or more consecutive days with Tmax exceeding the daily threshold for a reference period of 30 years^24^. Reference period from 1951–1980 is used in this study. The 90^th^ percentile of daily Tmax, centered on 31 day window is defined as the threshold. The magnitude of each heatwave in a year is the sum of magnitudes of consecutive days of a heatwave, with daily magnitude calculated as follows:

1 $${M}_{i}=\{\begin{array}{ll}\frac{{T}_{i}-{T}_{30y25p}}{{T}_{30y75p}-{T}_{30y25p}} & if\,{T}_{i} < {T}_{30y25p}\\ 0 & \,if\,{T}_{i}\le {T}_{30y25p}\end{array}$$

where, T_i_ is the Tmax on day ‘i’ of heatwave event, T_30y25p_ and T_30y75p_ are 25^th^ and 75^th^ percentile values of 30 year annual maximum temperature series within the reference period (1951–1980).

Meteorological droughts are defined based on Standardized Precipitation Index to characterize low rainfall events. Since droughts develop with the accumulation of rainfall deficits over some time period, averaging period of 1 month for computing SPI is selected. Statistically, the period of 1–24 months is considered best for estimating SPI and the index behaves erratically at shorter time scales^41^. India has a large variation of climate divided into seven climatic types: Arid, Semi-Arid, Tropical wet, Tropical wet and dry, Humid subtropical and Mountains. Single index value can’t capture the drought conditions in all the climatic zones of India. We might miss rainfall deficits of some regions by setting the threshold very high with the intention of focusing on extremely dry events. Therefore, drought events are defined below three different thresholds and classified into three categories: Moderate (SPI < −1.3), Severe (SPI < −1.6) and Extreme (SPI < −2).

### Concurrent droughts and heatwaves

Special report of Intergovernmental Panel on Climate Change (IPCC)^42^ defines compound events in three ways: a) simultaneous or successive occurrence of two or more extreme events b) combination of extreme events which can amplify the impact and c) combination of events which are not extreme at individual level but lead to an extreme event when combined. Monthly rainfall deficits and corresponding temperature extremes are the primary interests in this study. Concurrent extremes are defined as simultaneous exceedances of SPI-1 month and heatwaves for fixed thresholds.

### Extraction of spatial extent of concurrent droughts and heatwaves

One particular drought or heatwave event is not limited to single grid, but the same event spread over a large region. Spatial Extent is extracted by counting the number of grids affected by concurrent droughts and heatwaves which also avoids the multiple counting of same event at nearby grids. Fractional spatial extent is defined as the ratio of no. of grids affected due to concurrent droughts and heatwaves to the total no. of grids of India. Yearly values of fractional spatial extent are computed to understand the impact of concurrent extremes.

### Evaluating the change in the distribution

Two sample Cramér-von Mises test^34^ and Anderson-Darling test^36^ are used to compare the distributions of spatial extent during the period 1981–2010 with base period 1951–1980. Both the tests belong to the class of EDF (Empirical Distribution Function) statistics and they are competitive in terms of power^43^. They test the null that both the distributions are coming from same distribution. Let ${\hat{F}}_{m}(x)$ and Let ${\hat{F}}_{n}(x)$ be empirical CDFs of two samples, the Cramér-von Mises test statistic based on square of average distance between empirical distributions is given by

2 $${C}_{mn}={\int }_{-\infty }^{\infty }{|{\hat{F}}_{m}(x)-{\hat{F}}_{n}(x)|}^{2}dF(x)$$

Null hypothesis is rejected for large value of test statistic and what large means can be answered by characterizing the asymptotic distribution of test statistic^33,34^. The two sample Anderson-Darling test statistic^44,45^ is given by

3 $$\,{A}_{mn}^{2}=\frac{mn}{N}{\int }_{-\infty }^{\infty }\frac{{\{{F}_{m}(x)-{F}_{n}(x)\}}^{2}}{{H}_{N}(x)\{1-{H}_{N}(x)\}}d{H}_{N}(x)$$

where, $N=m+n$ and ${H}_{N}(x)=\{m{F}_{m}(x)+n{F}_{n}(x)\}/N$ is the distribution function of combined sample.

## Electronic supplementary material

Supplementary Information

## References

[1] Joseph PV, Bindu G, Preethi B (2016). Impact of the upper tropospheric cooling trend over central Asia on the Indian summer monsoon rainfall and the bay of Bengal cyclone tracks. Curr. Sci..

[2] Pai DS, Nair SA, Ramanathan AN (2013). Long term climatology and trends of heat waves over India during the recent 50 years (1961–2010). Mausam.

[3] Rohini P, Rajeevan M, Srivastava AK (2016). On the Variability and Increasing Trends of Heat Waves over India. Sci. Rep..

[4] Murari KK, Ghosh S, Patwardhan A, Daly E, Salvi K (2015). Intensification of future severe heat waves in India and their effect on heat stress and mortality. Reg. Environ. Chang..

[5] Kumar KK, Rajagopalan B, Hoerling M, Bates G, Cane M (2006). Unraveling the Mystery of Indian Monsoon Failure During El Nino. Science..

[6] De US, Mukhopadhyay RK (1998). Severe heat wave over the Indian subcontinent in 1998, in perspective of global climate. Curr. Sci..

[7] Ratnam JV, Behera SK, Ratna SB, Rajeevan M, Yamagata T (2016). Anatomy of Indian heatwaves. Sci. Rep..

[8] Dai A (2012). Increasing drought under global warming in observations and models. Nat. Clim. Chang..

[9] Christidis N, Jones GS, Stott PA (2015). Dramatically increasing chance of extremely hot summers since the 2003 European heatwave. Nat. Clim. Chang..

[10] Sun Y (2014). Rapid increase in the risk of extreme summer heat in Eastern China. Nat. Clim. Chang..

[11] Coumou D, Robinson A, Rahmstorf S (2013). Global increase in record-breaking monthly-mean temperatures. Clim. Chang..

[12] Fischer EM, Seneviratne SI, Lüthi D, Schär C (2007). Contribution of land-atmosphere coupling to recent European summer heat waves. Geophys. Res. Lett..

[13] Durre I, Wallace JM, Lettenmaier DP (2000). Dependence of extreme daily maximum temperatures on antecedent soil moisture in the contiguous United States during summer. J. Clim..

[14] Diffenbaugh NS, Pal JS, Giorgi F, Gao X (2007). Heat stress intensification in the Mediterranean climate change hotspot. Geophys. Res. Lett..

[15] Hao Z, AghaKouchak A, Phillips TJ (2013). Changes in concurrent monthly precipitation and temperature extremes. Environ. Res. Lett..

[16] AghaKouchak A, Cheng L, Mazdiyasni O, Farahmand A (2014). Global warming and changes in risk of concurrent climate extremes: Insights from the 2014 California drought. Geophys. Res. Lett..

[17] Mazdiyasni O, AghaKouchak A (2015). Substantial increase in concurrent droughts and heatwaves in the United States. Proc. Natl. Acad. Sci..

[18] Zscheischler, J. & Seneviratne, S. I. Dependence of drivers affects risks associated with compound events. *Sci*. *Adv*. **3** (2017). 10.1126/sciadv.1700263 PMC5489265 28782010

[19] Mallya G, Mishra V, Niyogi D, Tripathi S, G. R (2016). Trends and variability of droughts over the Indian monsoon region. Weather Clim. Extrem..

[20] Mishra A, Liu SC (2014). Changes in precipitation pattern and risk of drought over India in the context of global warming. J. Geophys. Res. Atmos..

[21] Zhang X, Obrin R, Wei C, Chen N, Niyogi D (2017). Droughts in India from 1981 to 2013 and Implications to Wheat Production. Sci. Rep..

[22] Perkins SE, Alexander LV, Nairn JR (2012). Increasing frequency, intensity and duration of observed global heatwaves and warm spells. Geophys. Res. Lett..

[23] Perkins SE, Alexander LV (2013). On the measurement of heat waves. J. Clim..

[24] Russo S (2015). heat wave Top ten European heatwaves since 1950 and their occurrence in the coming decades. Environ. Res. Lett..

[25] Mckee, T. B., Doesken, N. J. & Kleist, J. The relationship of drought frequency and duration to time scales. in *Eighth Conference on Applied Climatology* 179–184, American Meteorological Society, Boston, Massachusetts (1993).

[26] World Meteorological Organization. Standardized Precipitation Index User Guide. *WMO*-*No*. **1090**, Geneva (2012).

[27] Mann HB (1945). Nonparametric Tests AgainstTrend. Econom. Soc..

[28] Sen PK (1968). Estimates of the Regression Coefficient Based on Kendall’s Tau. J. Am. Stat. Assoc..

[29] Parthasarathy, B., Kumar, K. R. & Munot, A. Homogeneous regional summer monsoon rainfall over India: interannual variability and teleconnections. *Research Rep*. **70**, IITM, Pune (1996).

[30] Mishra V, Aadhar S, Asoka A, Pai S, Kumar R (2016). On the frequency of the 2015 monsoon season drought in the Indo-Gangetic Plain. Geophys. Res. Lett..

[31] Kumar KN, Rajeevan M, Pai DS, Srivastava AK, Preethi B (2013). On the observed variability of monsoon droughts over India. Weather Clim. Extrem..

[32] Cramér H (1928). On the composition of elementary errors. Scand. Actuar. J..

[33] Anderson TW, Darling DA (1952). Asymptotic Theory of Certain ‘Goodness of Fit’ Criteria Based on Stochastic Processes. Ann. Math. Stat..

[34] Anderson TW (1962). On the distribution of the two-sample Cramér-von Mises criterion. Ann. Math. Stat..

[35] Büning H (2002). Robustness and power of modified Lepage, Kolmogorov-Smirnov and Cramér-von Mises two- sample tests. J. Appl. Stat..

[36] Anderson TW, Darling DA (1954). A Test of Goodness of Fit. J. Am. Stat. Assoc..

[37] Barnett, V. & Lewis, T. Outliers in Statistical Data. John Wiley & Sons Ltd. (1978).

[38] Pai DS (2014). Development of a new high spatial resolution (0.25° × 0.25°) Long Period (1901-2010) daily gridded rainfall data set over India and its comparison with existing data sets over the region. Mausam.

[39] Srivastava A, Rajeevan M, Kshirsagar S (2009). Development of a high resolution daily gridded temperature data set (1969–2005) for the Indian region. Atmos. Sci. Lett..

[40] Gilleland, E. & Katz, R. W. extRemes 2.0: An Extreme Value Analysis Package in R. *J*. *Stat*. *Softw*. **72** (2016).

[41] Wu H, Svoboda MD, Hayes MJ, Wilhite A, Wen F (2007). Appropriate application of the Standardized Precipitation Index in arid locations and dry seasons. Int. J. Climatol..

[42] IPCC. Managing the risks of extreme events and disasters to advance climate change adaptation. A special report of working groups I and II of the Intergovernmental Panel on Climate Change. *IPCC*, Cambridge University Press, Cambridge (2012).

[43] Stephens MA (1974). EDF Statistics for Goodness of Fit and Some Comparisons. J. Am. Stat. Assoc..

[44] Darling DA (1957). The Kolmogorov-Smirnov, Cramer-von Mises Tests. Ann. Math. Stat..

[45] Pettitt AN (1976). A Two-Sample Anderson–Darling Rank Statistic. Biometrika.

