# Supplementary material for: Increasing frequency and spatial extent of concurrent meteorological droughts and heatwaves in India
Source: Sci Rep. 2017 Nov 14;7:15582. doi: 10.1038/s41598-017-15896-3 (PMC5686168; doi:10.1038/s41598-017-15896-3)
Supplement: Supplementary file 1 — Supplementary Information [file 41598_2017_15896_MOESM1_ESM.pdf]

Supplementary Information for:

Increasing frequency and spatial extent of concurrent meteorological droughts and heatwaves in India

Shailza Sharma[1], Pradeep Mujumdar[1,2,*]

[1]Department of Civil Engineering, Indian Institute of Science, Bangalore, 560012, India.

[2]Divecha Center for Climate Change, Indian Institute of Science, Bangalore, 560012, India.

[*]Corresponding Author, Email: pradeep@civil.iisc.ernet.in

Supplementary Figures

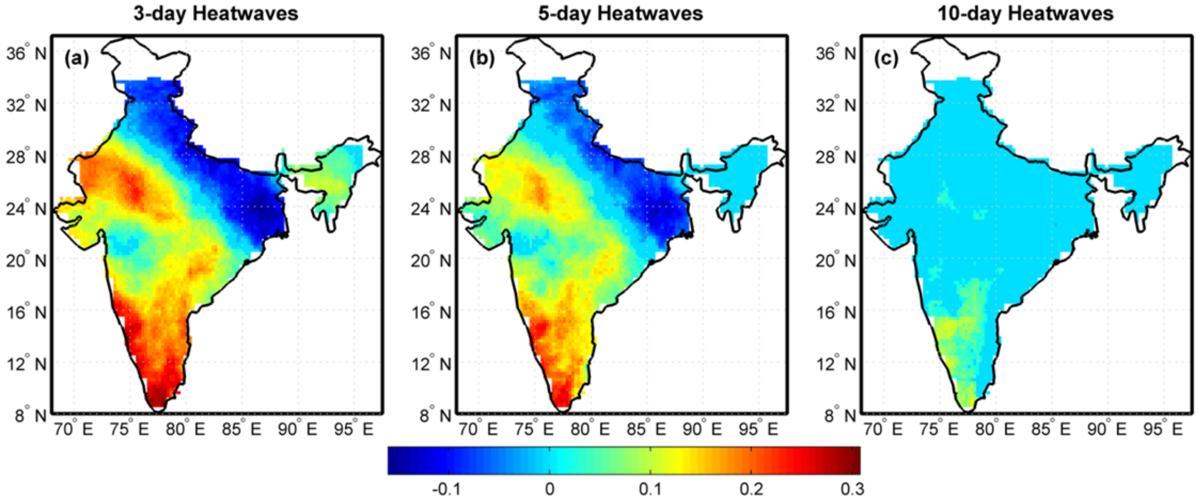

Figure S1. Trends in frequency of 90th percentile heatwaves over India for the period 1951-2010. (a) 3-day heatwaves show increasing trends in Northwest India, Peninsular India, some parts of West Central India and Northeast India, (b) 5-day heatwaves are affecting almost same regions as of 3-day heatwaves but magnitude of increase in the frequency is low compared to 3-day heatwaves and (c) 10-day heatwaves are increasing in lower Western Ghats and no trends are present in any other part of India. This figure is plotted in Matlab R2014a (Version 8.3.0.532, URL: https://in.mathworks.com).

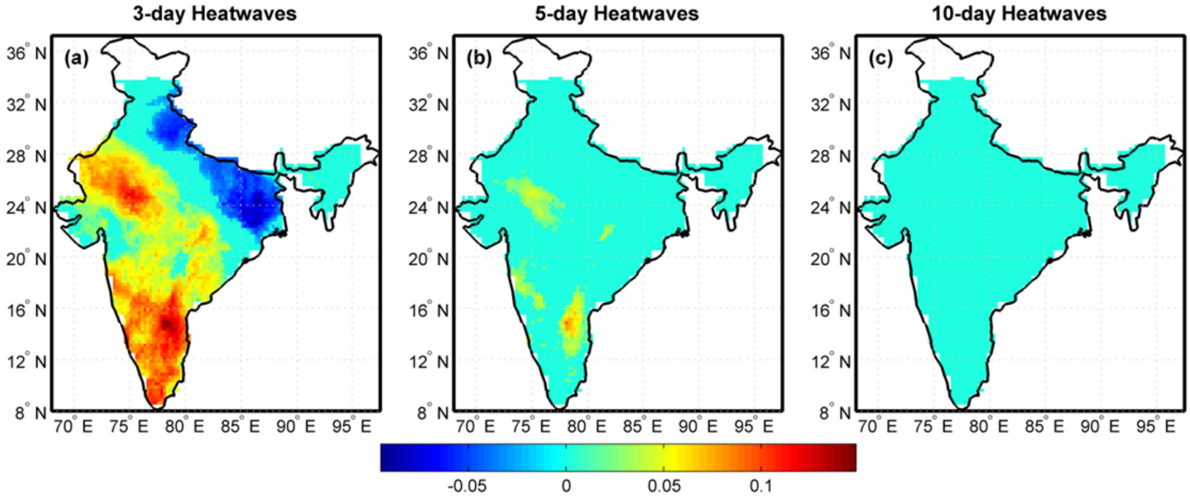

Figure S2. Trends in frequency of 95th percentile heatwaves over India during the period 1951-2010. (a) 3-day heatwaves are increasing in Northwest India, Peninsular India and some parts of West Central India, (b) 5-day heatwaves show increasing trends in small portions of Rajasthan, Maharashtra, Andhra Pradesh and Chhattisgarh, no trends are found anywhere else and (c) 10-day heatwaves have no trends in any part of India. This figure is plotted in Matlab R2014a (Version 8.3.0.532, URL: https://in.mathworks.com).

Table S1: Ranking of top five historical heat wave events of India based on the peaks of Heatwave Magnitude Index daily (HWMId)

Year	Location	HWMId Peak	Duration of the event (days)
1983	12.5N, 76.5E	75.1	63
1998	8.5N, 73.5E	55.8	38
2003	14.5N, 78.5E	33.5	19
1979	28.5N, 88.5E	29.7	16
1973	16.5N, 75.5E	28.8	26

Table S2: Ranking of top five historical heat wave events of India based on the spatial extent

Year	% Area HWMId >=6	% Area HWMId >=9	% Area HWMId >=15	% Area HWMId >=24
1998	48.7	34.3	8.7	3.0
1995	46.0	33.4	10.1	0
2010	37.3	26.3	7.5	0.6
1988	33.7	16.7	0.9	0
1973	33.4	13.7	4.5	0.6

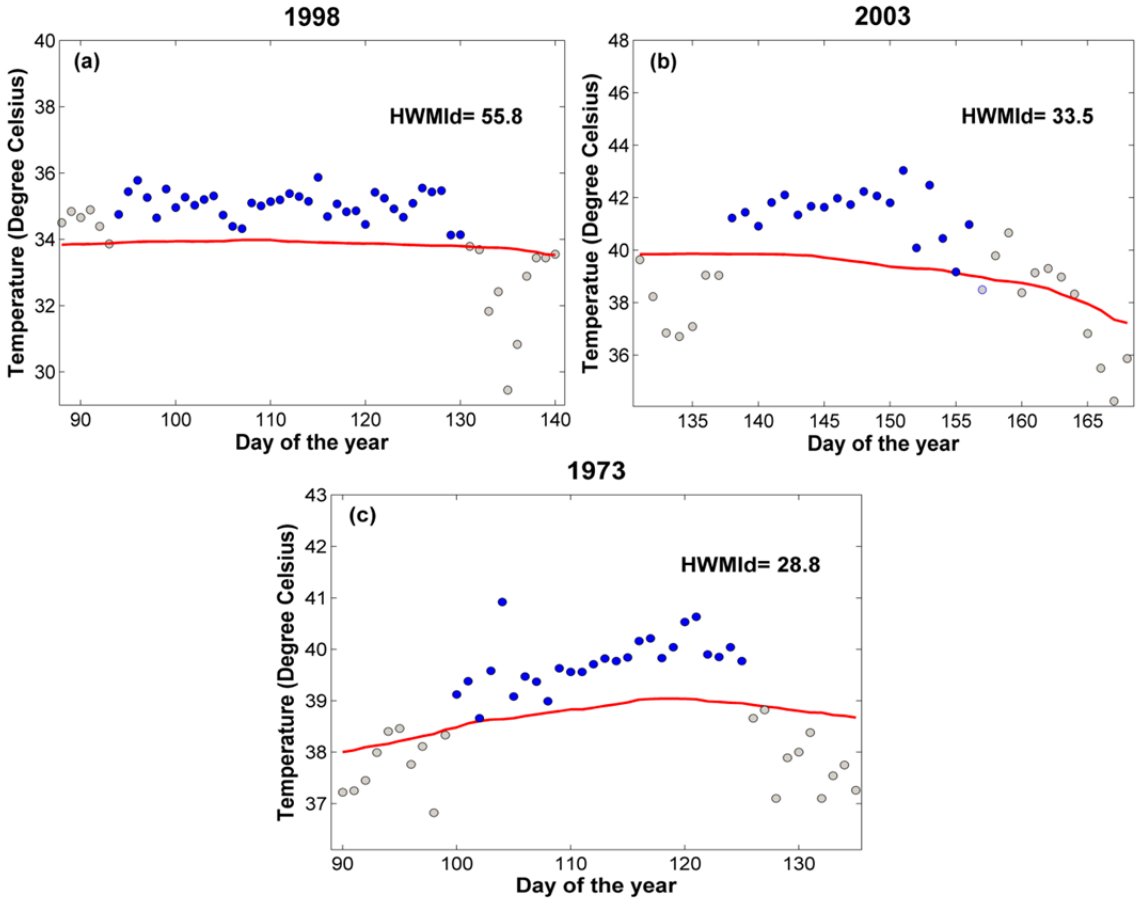

Figure S3. Time series of major heatwaves in India since 1950 at locations where peak of HWMId is observed. The time series of (a) 1998 heatwave event at 12.5N, 76.5E (b) 2003 heatwave event at 14.5N, 78.5E and (c) 1973 heatwave event at 14.5N, 78.5E. Grey circles are daily Tmax values and the blue circles are the days composing heatwave. Total number of blue circles represents the duration of heatwave event. Beginning day and end day of the heatwave are marked on x-axis. The red line is daily temperature threshold, which is estimated using 30 year reference period (1951-1980). The figure is created in Matlab R2014a (Version 8.3.0.532, URL: https://in.mathworks.com).

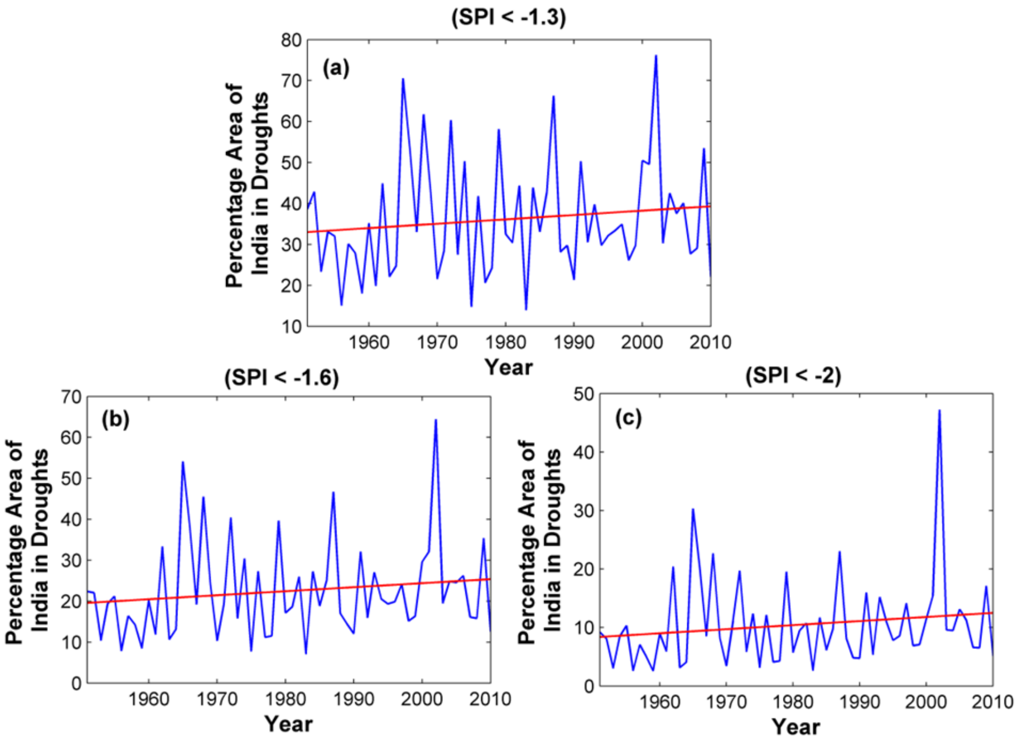

Figure S4. Yearly percentage of India in meteorological droughts defined for three different thresholds (a) SPI < -1.3 (b) SPI < -1.6 and (c) SPI < -2. No trends are observed in yearly spatial area of India affected by droughts across whole India. This figure is plotted in Matlab R2014a (Version 8.3.0.532, URL: https://in.mathworks.com).

Table S3. Non-parametric Mann-Kendall trend test results at 5% significance level for percentage area of India in droughts

Thresholds for droughts	Test Statistic	p-value	Interpretation against H_0	Conclusion
SPI < -1.3	0.0965	0.2835	No evidence	Monotonic trends are absent
SPI < -1.6	0.1240	0.1676	No evidence	
SPI < -2	0.1490	0.0967	No evidence	

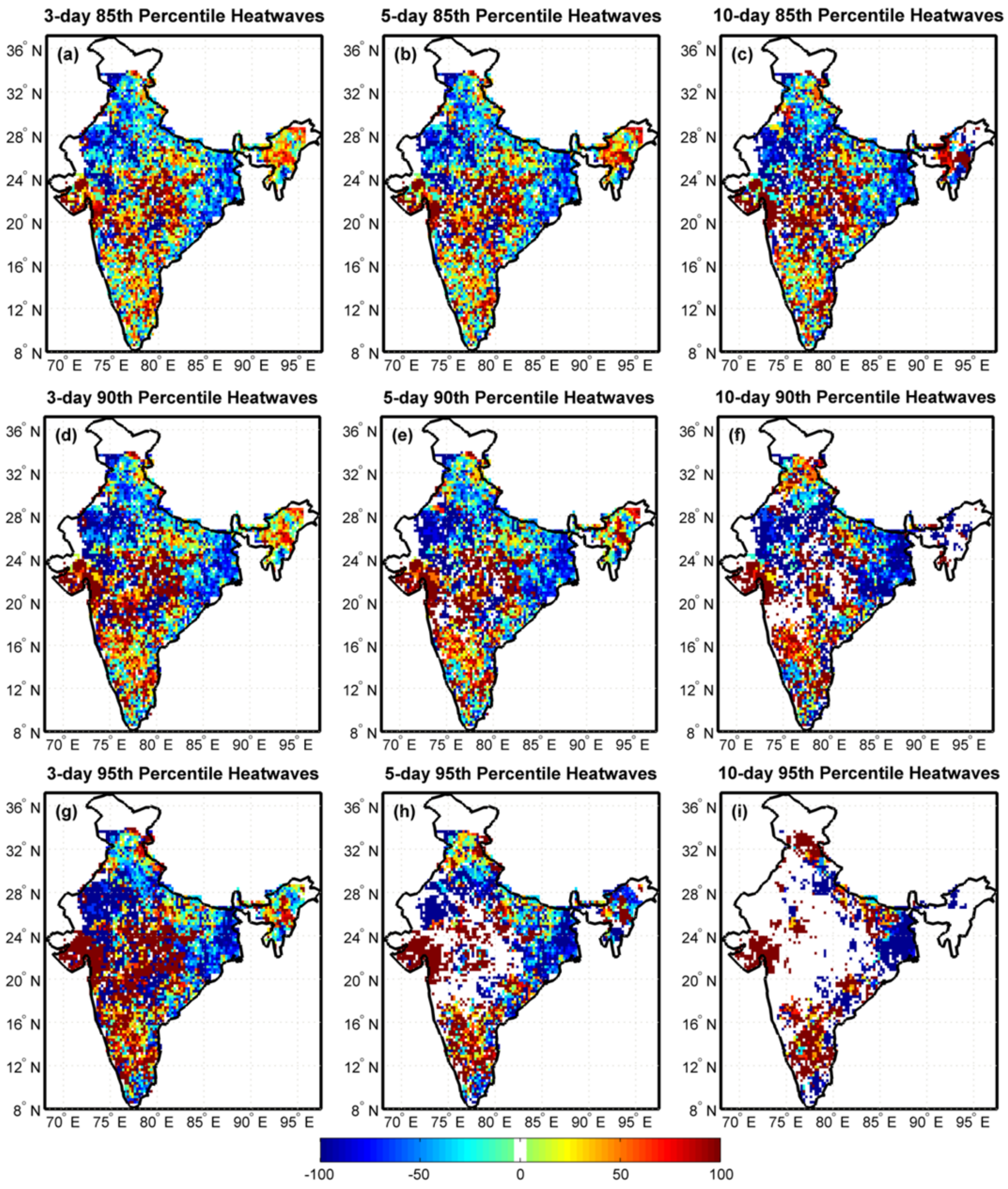

Figure S5. Percentage changes in concurrent severe droughts defined below SPI < -1.6 and heatwaves during 1981-2010 with respect to the base period 1951-1980. These maps are generated in Matlab R2014a (Version 8.3.0.532, URL: https://in.mathworks.com).

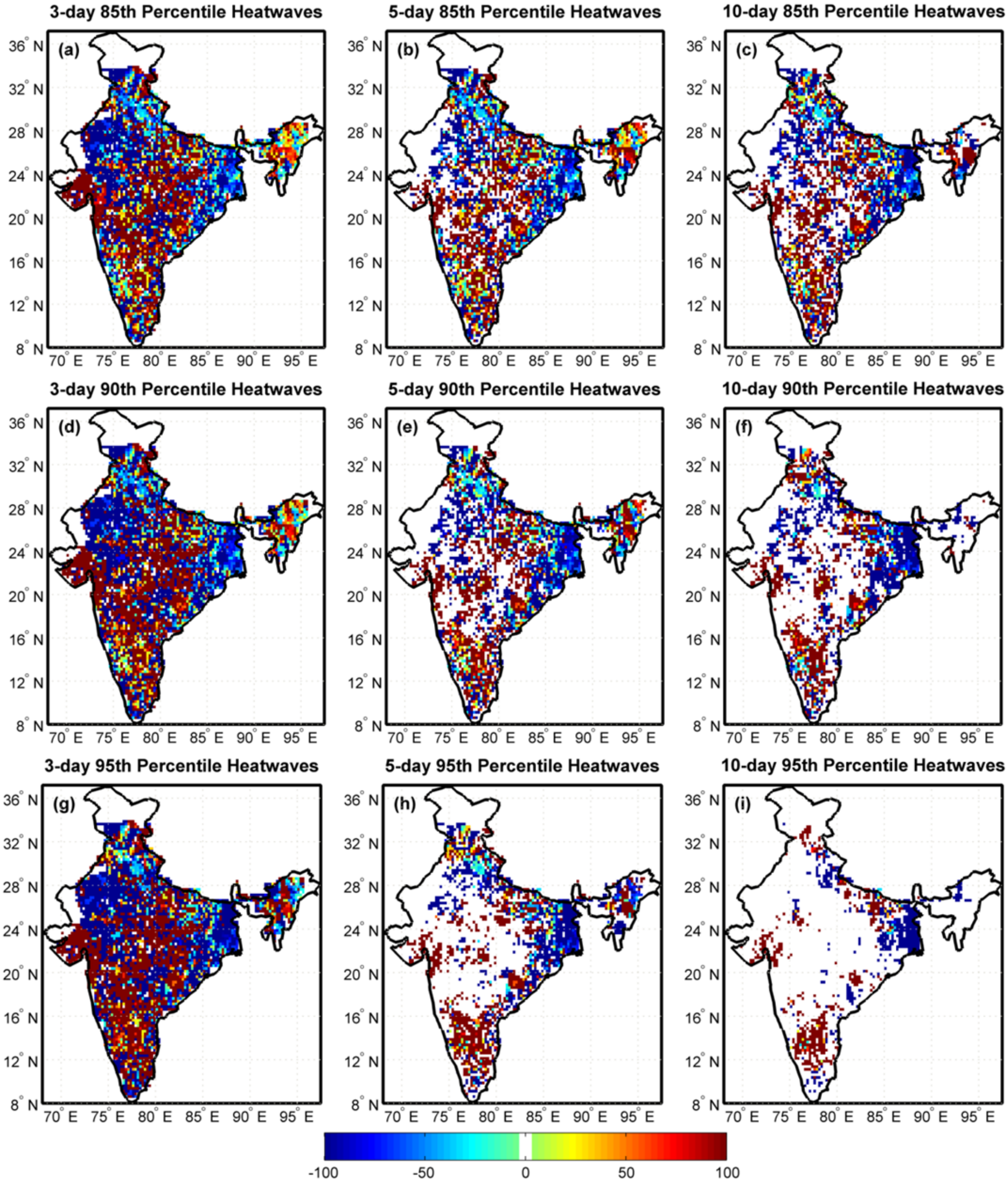

Figure S6. Percentage changes in concurrent extreme droughts defined below SPI < -2 and heatwaves during 1981-2010 with respect to the base period 1951-1980. These maps are generated in Matlab R2014a (Version 8.3.0.532, URL: https://in.mathworks.com).

(SPI < -1.3)

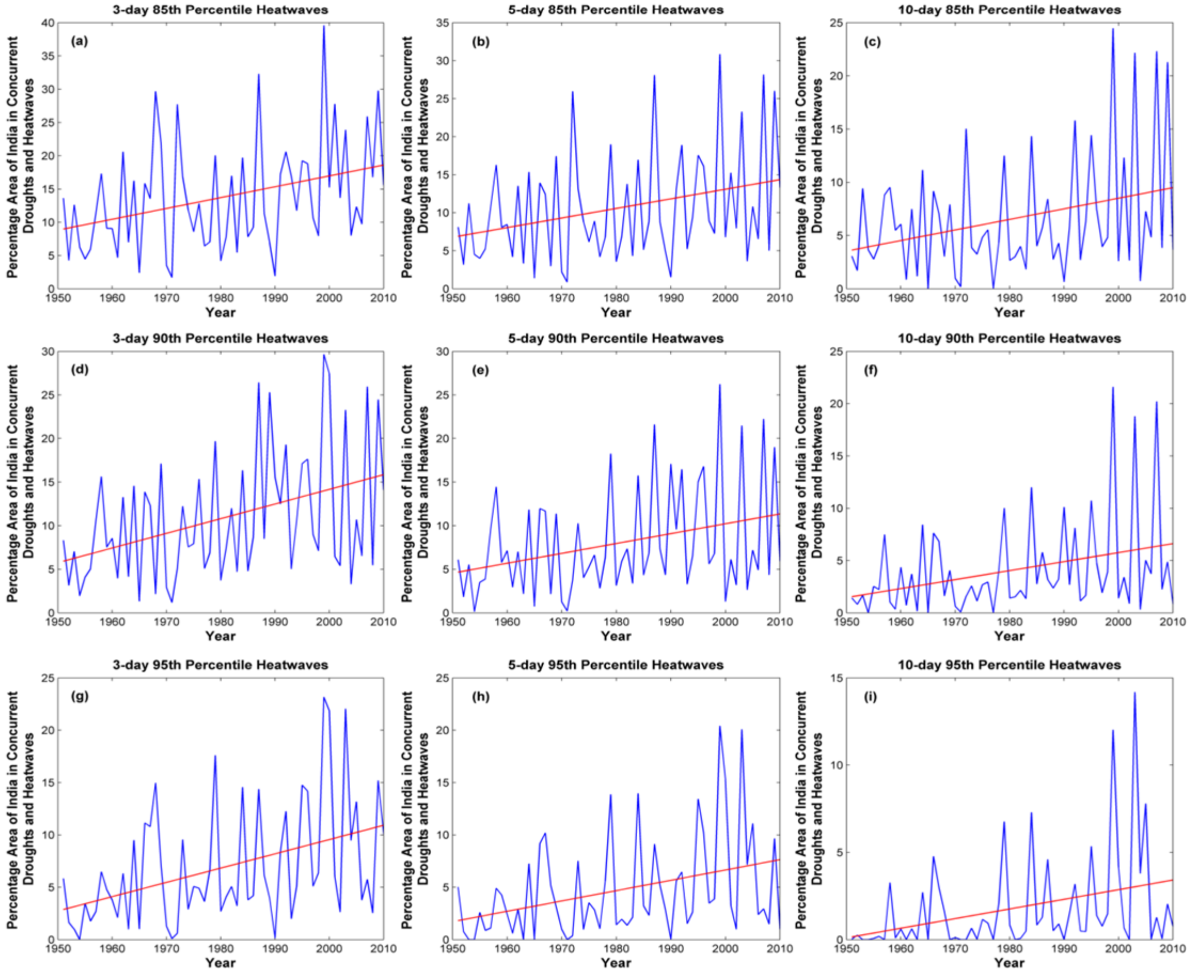

Figure S7. Trends in percentage area of India in concurrent moderate droughts (SPI < -1.3) and heatwaves over the period 1951-2010. This figure is plotted in Matlab R2014a (Version 8.3.0.532, URL: https://in.mathworks.com).

Table S4. Non-parametric Mann-Kendall trend test results at 5% significance level for percentage area of India in concurrent moderate droughts (SPI < -1.3) and heatwaves

<table>
<tr><th></th><th>Test Statistic</th><th>p-value</th><th>Interpretation against H_0</th><th>Conclusion</th></tr>
<tr><td>3-day 85th Percentile HW</td><td>0.228</td><td>0.0102</td><td rowspan="9">Reject H_0</td><td rowspan="9">Monotonic trend is present</td></tr>
<tr><td>3-day 90th Percentile HW</td><td>0.256</td><td>0.0040</td></tr>
<tr><td>3-day 95th Percentile HW</td><td>0.284</td><td>0.0014</td></tr>
<tr><td>5-day 85th Percentile HW</td><td>0.182</td><td>0.0406</td></tr>
<tr><td>5-day 90th Percentile HW</td><td>0.190</td><td>0.0321</td></tr>
<tr><td>5-day 95th Percentile HW</td><td>0.252</td><td>0.0047</td></tr>
<tr><td>10-day 85th Percentile HW</td><td>0.116</td><td>0.0047</td></tr>
<tr><td>10-day 90th Percentile HW</td><td>0.206</td><td>0.0190</td></tr>
<tr><td>10-day 95th Percentile HW</td><td>0.278</td><td>0.0206</td></tr>
</table>

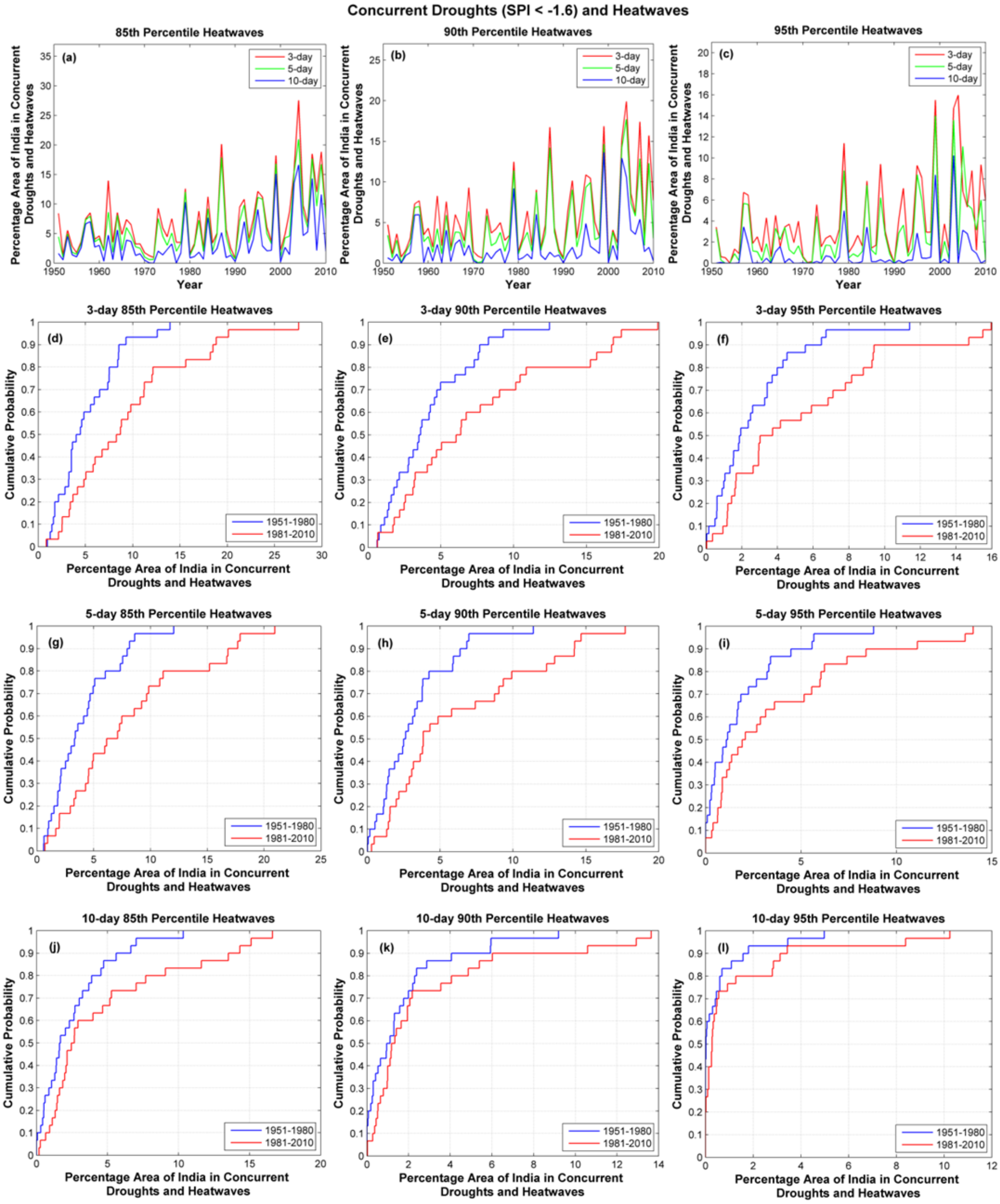

Figure S8. Spatial area of India in concurrent severe droughts defined below SPI < -1.6 and heatwaves. This figure is plotted in Matlab R2014a (Version 8.3.0.532, URL: https://in.mathworks.com).

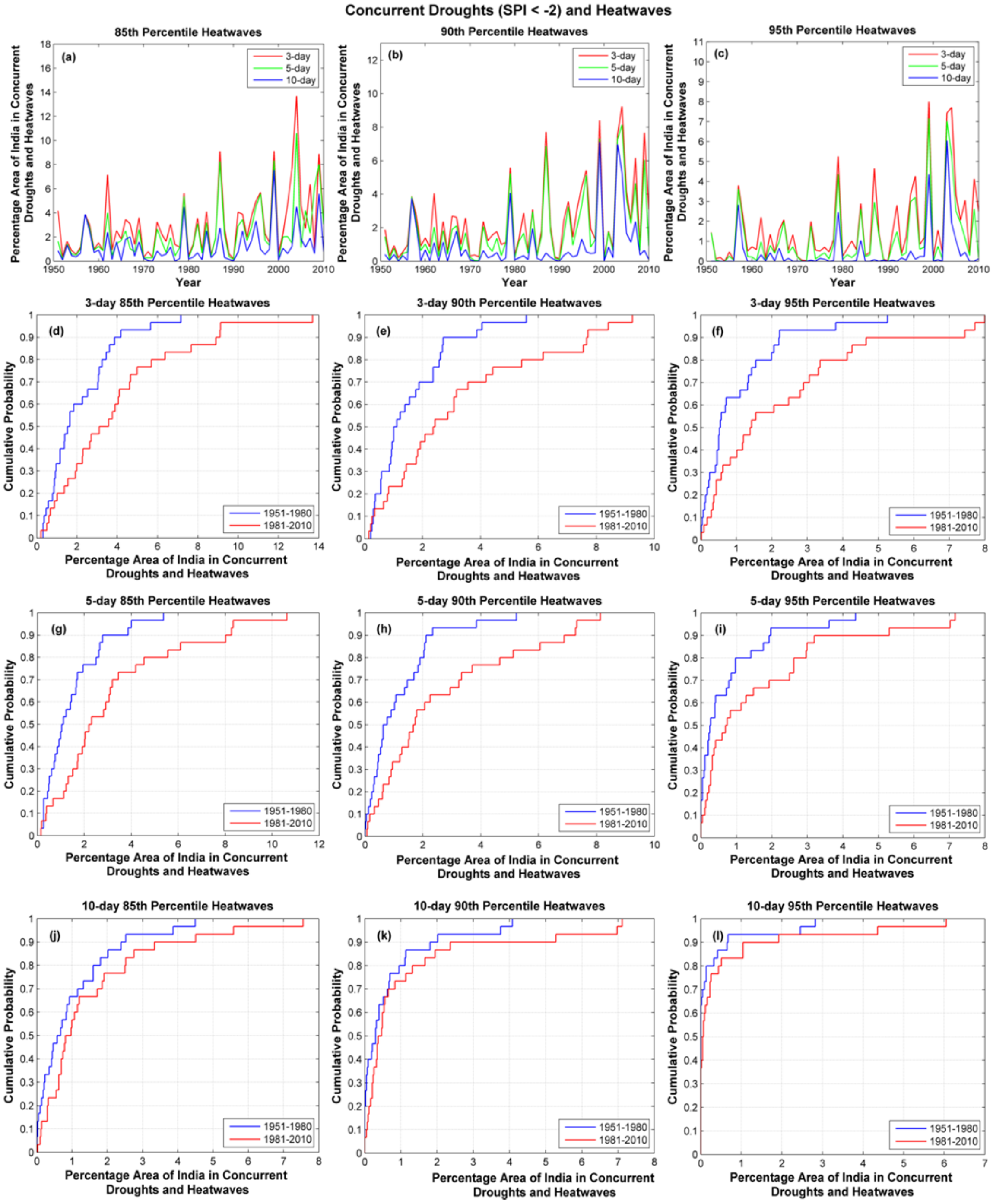

Figure S9. Spatial area of India in concurrent extreme droughts defined below SPI < -2 and heatwaves. This figure is plotted in Matlab R2014a (Version 8.3.0.532, URL: https://in.mathworks.com).

(SPI < -1.3)

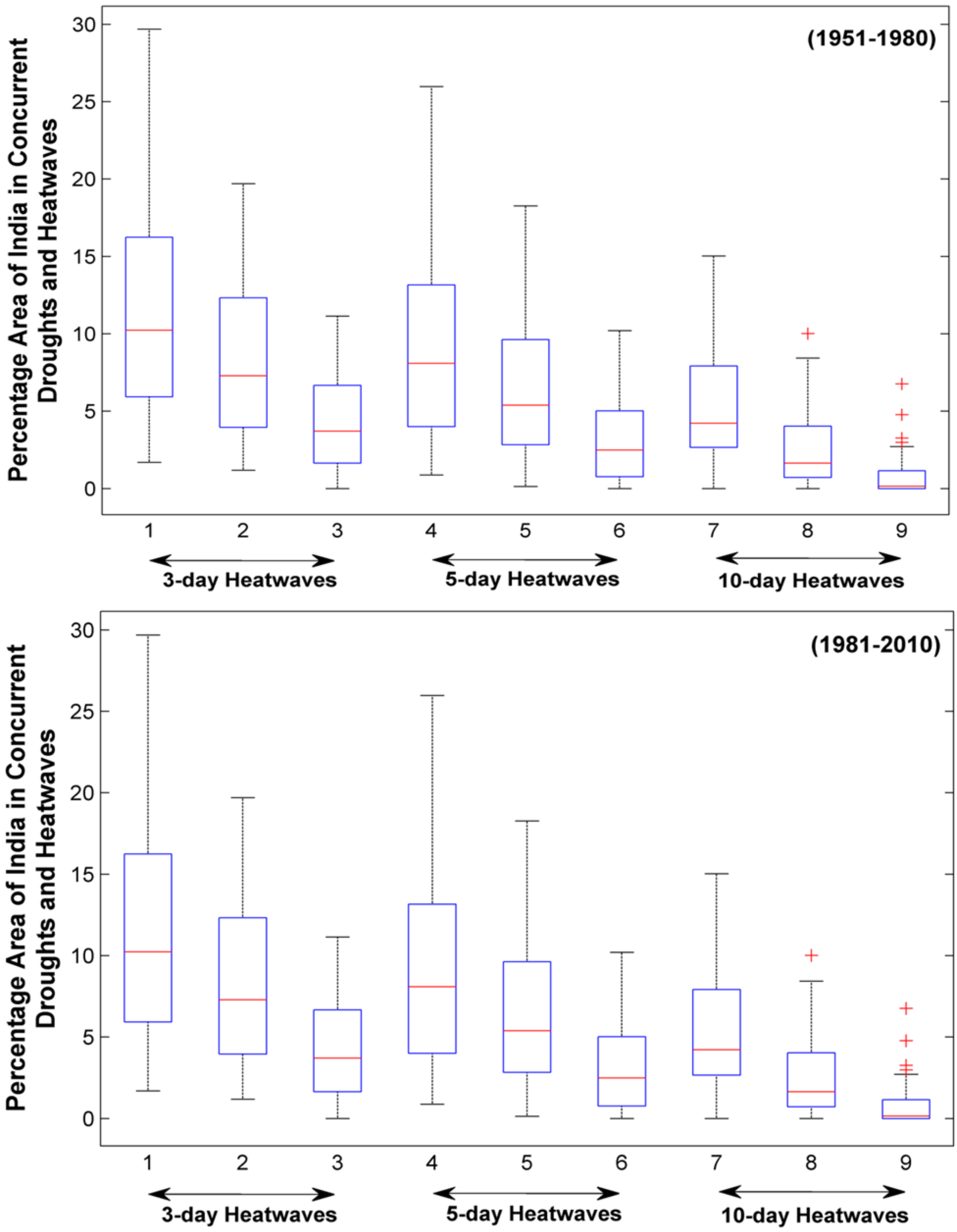

Figure S10. Box plots to detect outliers in percentage area of India affected by concurrent moderate droughts (SPI < -1.3) and heatwaves.

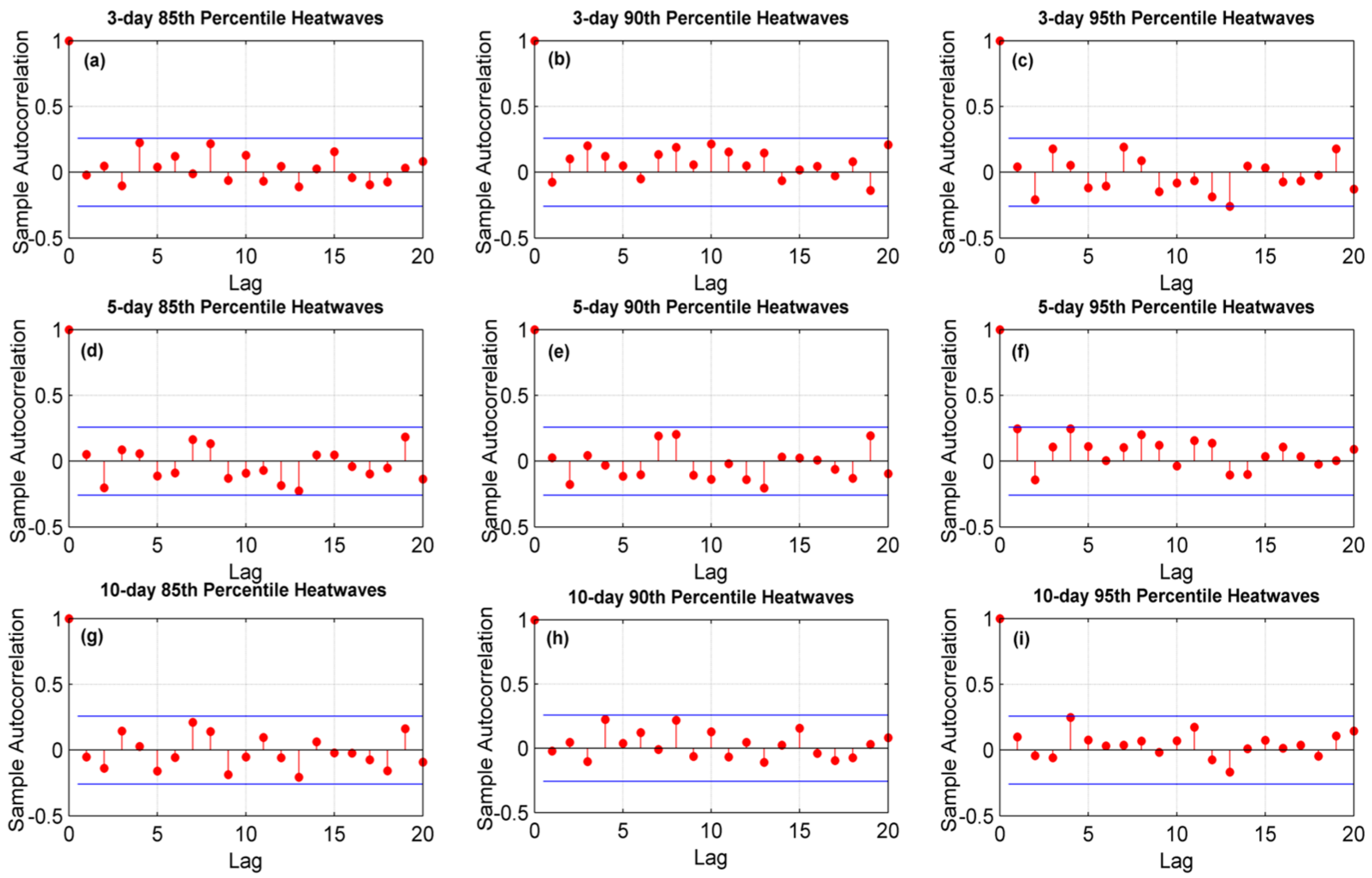

Figure S11. Sample autocorrelation of percentage area of India for concurrent moderate droughts (SPI < -1.3) and heatwaves. All the ACF values are within confidence bounds.
